# Design of a single-arm clinical trial of regenerative therapy by periurethral injection of adipose-derived regenerative cells for male stress urinary incontinence in Japan: the ADRESU study protocol

**DOI:** 10.1186/s12894-017-0282-7

**Published:** 2017-09-25

**Authors:** Shinobu Shimizu, Tokunori Yamamoto, Shinobu Nakayama, Akihiro Hirakawa, Yachiyo Kuwatsuka, Yasuhito Funahashi, Yoshihisa Matsukawa, Keisuke Takanari, Kazuhiro Toriyama, Yuzuru Kamei, Kazutaka Narimoto, Tomonori Yamanishi, Osamu Ishizuka, Masaaki Mizuno, Momokazu Gotoh

**Affiliations:** 1 Center for Advanced Medicine and Clinical Research, Nagoya University Hospital, 65 Tsurumai-cho, Showa-ku, Nagoya, Aichi 466-8560 Japan; 2 Department of Urology, Nagoya University Graduate School of Medicine, 65 Tsurumai-cho, Showa-ku, Nagoya, Aichi 466-8550 Japan; 3 Department of Plastic and Reconstructive Surgery, Nagoya University Graduate School of Medicine, 65 Tsurumai-cho, Showa-ku, Nagoya, Aichi 466-8550 Japan; 4 Department of Plastic and Reconstructive Surgery, Nagoya City University Hospital, 1-Kawasumi, Mizuho-cho, Mizuho-ku, Nagoya, Aichi 467-8602 Japan; 5 Department of Integrative Cancer Therapy and Urology, Kanazawa University Graduate School of Medical Sciences, 13-1 Takara-machi, Kanazawa, Ishikawa 920-8640 Japan; 6 Department of Urology, Continence Center, Dokkyo Medical University, 880 Kita-Kobayashi, Mibu-machi, Shimotsuga-gun, Tochigi, 321-0293 Japan; 7 Department of Urology, Shinshu University School of Medicine, 3-1-1 Asahi, Matsumoto, 390-8621 Japan; 8 Department of Clinical Research Management, Clinical Research Center, National Hospital Organization Nagoya Medical Center, 4-1-1, Sannomaru, Naka-ku, Nagoya, Aichi 460-0001 Japan; 9 Department of Biostatistics and Bioinformatics, Graduate School of Medicine, The University of Tokyo, 7-3-1 Hongo, Bunkyo-ku, Tokyo, 113-0033 Japan

**Keywords:** Stress urinary incontinence, Lower urinary tract symptoms, Regenerative medicine, Cell therapy, Prostatectomy, Adipose-derived regenerative cells

## Abstract

**Background:**

Male stress urinary incontinence is a prevalent condition after radical prostatectomy. While the standard recommendation for the management of urine leakage is pelvic floor muscle training, its efficacy is still unsatisfactory. Therefore, we have focused on regenerative therapy, which consists of administering a periurethral injection of autologous regenerative cells from adipose tissue, separated using the Celution® system. Based on an interim data analysis of our exploratory study, we confirmed the efficacy and acceptable safety profile of this treatment. Accordingly, we began discussions with Japanese regulatory authorities regarding the development of this therapy in Japan. The Ministry of Health, Labour and Welfare suggested that we implement a clinical trial of a new medical device based on the Pharmaceutical Affaires Act in Japan. Next, we discussed the design of this investigator-initiated clinical trial (the ADRESU study) aimed at evaluating the efficacy and safety of this therapy, in a consultation meeting with the Pharmaceuticals and Medical Device Agency.

**Methods:**

The ADRESU study is an open-label, multi-center, single-arm study involving a total of 45 male stress urinary incontinence patients with mild-to-moderate urine leakage persisting more than 1 year after prostatectomy, in spite of behavioral and pharmacological therapies. The primary endpoint is the rate of patients at 52 weeks with improvement of urine leakage volume defined as a reduction from baseline greater than 50% by 24-h pad test. Our specific hypothesis is that the primary endpoint result will be higher than a pre-specified threshold of 10%.

**Discussion:**

The ADRESU study is the first clinical trial of regenerative treatment for stress urinary incontinence by adipose-derived regenerative cells using the Celution® system based on the Japanese Pharmaceutical Affaires Act. We will evaluate the efficacy and safety in this trial to provide an adequate basis for marketing approval with the final objective of making this novel therapy widely available for Japanese patients.

**Trial registration:**

This trial was registered at the University Hospital Medical information Network Clinical Trial Registry (UMIN-CTR Unique ID: UMIN000017901; Registered July 1, 2015) and at ClinicalTrials.gov (ClinicalTrials.gov Identifier: NCT02529865; Registered August 18, 2015).

## Background

Male stress urinary incontinence (SUI) is a secondary intrinsic sphincter deficiency and a prevalent condition after radical prostatectomy [1–5]. Individuals with SUI may limit activities of daily living to reduce the chance of urine leakage; hence, SUI can have a major impact on the quality of life (QOL) of patients. The standard therapy of male SUI is pelvic floor muscle training, and clenbuterol hydrochloride, which is solely approved for SUI indication in Japan; however, the efficacy of these treatments is still unsatisfactory. Another potential therapy is periurethral injection of collagen, but its efficacy has only been shown in short-term trials [6, 7], and this treatment is not currently approved for SUI in Japan. Another potential alternative involves the implantation of an artificial urinary sphincter, which is recommended for patients with severe incontinence [8–10]. Unfortunately, according to a Japanese report from the Office of Pharmaceutical Industry Research (OPIR News No.45), new pharmaceutical agents for SUI have not been developed in Japan as of 2015.

Meanwhile, we have focused on regenerative cell therapy of urethral sphincter deficiency based on several experimental studies [11–16]. We previously confirmed that periurethral injection of cultured adipose-derived stem cells improved leak point pressure in an SUI animal model [17]. Therefore, we planned a preliminary clinical study with the objective of developing a novel treatment using autologous adipose-derived regenerative cells (ADRCs) without cell culture. In other words, the ADRCs are isolated in a few hours by the Celution® system (Cytori Therapeutics, Inc., San Diego, CA, USA). That study was approved by the Ethics Committee of the Nagoya University Graduate School of Medicine, and also by the committee of the Japanese Ministry of Health, Labour and Welfare (MHLW) according to the Guidelines on Clinical Research using Human Stem Cells [18], and registered at the University Hospital Medical information Network Clinical Trial Registry (UMIN-CTR Unique ID: UMIN000006116). Based on an interim data analysis of that exploratory clinical study, we obtained preliminary evidence confirming that the periurethral injection of isolated ADRCs is effective and safe for use in the male SUI patients [19, 20]. However, the Celution® system is not currently available for treatment purposes in Japan. This system is only currently approved for use in clinical research under the Japanese ‘Act on the Safety of Regenerative Medicine’. Therefore, as our objective is to disseminate this novel therapy throughout Japan as soon as possible, we prepared a confirmatory study protocol and began discussions with the Japanese regulatory authorities to build a bridge from ‘benchside to bedside to community’ in cooperation with Cytori Therapeutics, Inc.

In this article, we provide the detailed design of this investigator-initiated clinical trial in male SUI patients as a pivotal study for the licensure of this therapy in Japan (the ADRESU study). Incidentally, the main design of this trial is undergoing the process of approval by the Pharmaceuticals and Medical Device Agency (PMDA), which is responsible for reviewing new pharmaceuticals, medical devices, and regenerative medicines; however, whether the final approval by PMDA is granted or not will depend on the data obtained in this study.

## Methods/Design

### Overall design and objective

The primary objective of this trial is to evaluate the efficacy and safety of periurethral injection of autologous ADRCs separated with the Celution® system in male SUI patients. The ADRESU study is an open-label, multi-center, single-arm study involving a total of 45 male patients with stress urinary incontinence, with mild-to-moderate urine leakage persisting more than 1 year after prostatectomy that achieved insufficient symptom improvement by behavioral and pharmacologic therapies. The primary endpoint of this study is the rate of patients with improvement in urine leakage volume. Our specific hypothesis is that the rate of patients with improvement in urine leakage volume, defined as a reduction greater than 50% from baseline by 24-h pad test, is higher than a pre-specified threshold of 10%. Additionally, we will consider whether or not patients with symptom improvement will also show improvement in QOL scores.

### Selection of subjects

First, we decided to select male patients with SUI as our patient population, not only because urinary incontinence mechanisms differ by sex, but also because few female data were available in our former study. Next, we considered prior incontinence history, in other words, duration and severity of incontinence after surgery. Regarding the duration of incontinence, there is enough evidence to show that patients who develop post-prostatectomy incontinence persisting for a period longer than 1 year are unlikely to recover function thereafter [1–5]. With respect to incontinence severity, we restricted our population to patients with mild-to-moderate urinary incontinence based on subgroup analysis of our available data in comparison with more severe patients. The mean reduction rate of leakage volume at 12 months of patients with mild-to-moderate and severe urinary incontinence was 62.6% and 18.0%, respectively (Table 1).

**Table 1:** Reduction rate of urine leakage volume from baseline by 24-h pad test in exploratory study

	Daily urine leakage volume at baseline	Reduction rate of daily urine leakage volume			
		1 month	3 months	6 months	12 months
Severe SUI (*n* = 5)	531.1 ± 229.2 g	−40.0 ± 62.6%	−6.2 ± 56.2%	2.9 ± 58.3%	18.0 ± 46.9%
Mild-to-moderate SUI (*n* = 9)	85.2 ± 55.0 g	17.0 ± 66.1%	32.2 ± 54.7%	27.6 ± 50.5%	62.6 ± 19.0%

Each data point represents the mean ± SD

The detailed inclusion criteria are as follows:

1. Males with stress urinary incontinence persisting more than 1 year after either of the following surgical procedures, with insufficient improvement of symptoms by behavioral and pharmacological therapies:
  i. Patients with symptoms after radical prostatectomy for localized prostate cancer and currently without relapse/metastasis, and prostate specific antigen (PSA) level less than 0.1 ng/mL for over 1 year
  ii. Patients with symptoms after transurethral prostatectomy or laser prostatectomy for prostatic hyperplasia and PSA level less than 4.0 ng/mL over 1 year
2. Age of 20 years or above
3. Mild-to-moderate urinary incontinence by the 24-h pad test
4. Patients who can keep a bladder diary in a satisfactory manner
5. Patients that can provide a signed informed consent

The main exclusion criteria are as follows:

1. Concurrent with any other types of urinary incontinence
2. History of urinary or reproductive surgery within 6 months
3. History of behavioral therapy or pharmacotherapy initiation within 3 months
4. Concurrent with diabetes insipidus
5. History of radiotherapy in the lower urinary tract
6. History of ADRCs treatment for stress urinary incontinence
7. History of any type of cell therapy within 6 months
8. Participation in any other clinical trial within 3 months
9. Concurrent with lower urinary tract obstruction
10. Concurrent with urolithiasis, urinary tract infection or interstitial cystitis
11. History of recurrent urinary tract infection
12. History or suspicion of malignant neoplasm within the last 5 years
13. Any other patients whom the trial investigator deems ineligible for this study

### Selection of patients for the control group

In general, a concurrent control group is needed for a clinical trial as the most common clinical trial designs for a confirmatory trial is a randomized, controlled, parallel-group design [21, 22]. Potential candidates for the control group include those receiving approved treatment (pharmaceuticals, behavioral therapy, or surgical procedure), placebo, sham treatment, or no treatment.

Regarding approved treatments, as shown in ‘*Selection of subjects*’, there is no conventional therapy in Japan for patients eligible for this study. To select placebo or sham treatment as a control group is not acceptable for clinical and ethical reasons given the invasive nature of the periurethral injection and liposuction. While a no-treatment group may be effective as a potential comparator, such a comparison is not always necessary because patients with post-prostatectomy incontinence persisting for 1 year are unlikely to improve from 1 year onward [1–5]. Therefore, we decided to implement a confirmatory single-arm study.

### Registration and informed consent

Patients are registered temporarily as candidates for this study after providing written informed consent and the investigators have confirmed that they meet most of the eligibility criteria. Thereafter, an investigator reviews the patient’s urination diary, the method of data collection, that is that the patient measures the weight of the urination pad, the number of incontinence episodes, and the number of pads used, and then records it at home for 7 consecutive days. Then, these data are assessed to determine eligibility by the inclusion criteria no. 3. After all eligibility criteria are confirmed, the patients are enrolled via electronic data capture system (Viedoc™, PCG Solutions Ab. Uppsala, Sweden), with the subsequent initialization of the treatment process.

### Preparation and injection of ADRCs

About 250 to 300 mL and an additional 20 to 30 mL of adipose tissue are manually suctioned from subcutaneous layer in abdominal wall or hip under general or spinal anesthesia. About 250 to 300 mL of extracted adipose tissue are applied into the Celution® system. This system is an apparatus designed to isolate ADRCs from human suctioned fat semi-automatically in 1 to 2 h. Collected ADRCs by the Celution® system are composed of a heterogeneous cell population, including adipose-derived stem cells, endothelial (progenitor) cells and vascular smooth muscle cells [23]. Finally, we will obtain a 5-mL ADRCs solution including about 1 × 10^7^ nucleated cells.

For periurethral injection, we will prepare two materials: one consists of 1 mL of ADRCs solution for direct injection into the sphincter, and the other is a 20 mL mixture comprising 4 mL of ADRCs solution with 16 mL of fat for injection into the submucosal spaces. A ‘NUU Device’™ (Hakko Co. Ltd. Bunkyo-ku, Tokyo, Japan), which is a puncture needle of 38 cm in length, is inserted through the endoscope into the urethra under direct endoscopic vision. Initially, 1 mL of ADRC solution are injected using the 22 G puncture needle to a 5- to 10-mm depth into the rhabdosphincter at 5 and 7 o’clock positions. Subsequently, a 20-mL mixture of ADRCs and fat is administered with an 18 G puncture needle to about a 10-mm depth into the submucosal spaces at the 4 and 8 o’clock positions (if needed 6 o’clock position) at the external urethral sphincter area. Detailed procedures for the preparation and injection of ADRC solutions are provided in previous reports [19, 20].

### Response variables (outcomes)

Patients will be followed-up for 52 weeks after injection of ADRCs. The ADRESU study schedule is shown in Table 2.

**Table 2:** The ADRESU study data collection schedule

	Screening period		Operation	Observation period							
	Provisional registration	Registration	Day 0	Day 1	At end of hospitalization	Week 2	Week 4	Week 12	Week 26	Week 38	Week 52
Eligibility criteria	X	X									
Vital signs^a^			X	X	X	X					
Oxygen saturation			X	X	X	X					
Laboratory tests											
Infections^b^		X									
Hematology^c^, Biochemistry^d^, CRP, Coagulation^e^, Urinalysis^f^		X		X	X	X	X	X	X		X
PSA		X						X	X		X
12 Lead electrocardiography		X		X	X	X					
Chest X-rays		X									
Urination diary^g^		X				X	X	X	X	X	X
QOL scores^h^		X							X		X
Patient overall satisfaction		X							X		X
Urodynamic parameters^i^		X				X	X	X	X		X
Transrectal ultrasonography		X						X	X		X
MRI		X				X			X		X
Liposuction			X								
Periurethral injection of ADRCs, and mixture of ADRCs and fat			X								
Concomitant therapies	X	X	X	X	X	X	X	X	X	X	X
Adverse events	X	X	X	X	X	X	X	X	X	X	X

^a^ Blood pressure, pulse rate, body temperature

^b^ HBs antigen, HCV antibody, HIV antibody, serologic test of syphilis

^c^ Red blood, hemoglobin, hematocrit, white blood cell count, fraction of leucocytes (basophil, eosinophil, neutrophil, lymphocyte, monocyte), platelet count

^d^ Total protein, albumin, total cholesterol, blood urea nitrogen, creatinine, uric acid, sodium, chloride, potassium, calcium, phosphate, lactate dehydrogenase, aspartate aminotransferase, alanine aminotransferase, alkaline phosphatase, gamma-glutamyl transferase, total bilirubin, creatinine kinase

^e^ Prothrombin time, activated partial thromboplastin time, fibrinogen

^f^ pH, protein, glucose, urobilinogen, occult blood

^g^ Urine leakage volume, number of incontinence episodes, number of pads used

^h^ International Consultation on Incontinence Questionnaire-Short Form, King’s Health Questionnaire

^i^ Maximum urethral closing pressure, functional profile length, abdominal leak point pressure

*Abbreviations: CRP* C-reactive protein, *PSA* Prostate-Specific Antigen, *QOL* quality of life, *MRI* magnetic resonance imaging, *ADRCs* adipose-derived regenerative cells

As a general rule, the primary endpoint should reflect clinically relevant and meaningful effects [24]. The USA Food and Drug Administration (FDA), which is the regulatory authority of the USA, recommends commonly used effectiveness endpoints for SUI listed as follows: amount of urine leakage (1-h pad weight test, 24-h pad test), number of incontinence episodes, number of pads used, QOL, and urodynamic measurements [25]. However, the FDA insists that dryness is the ultimate goal of treatment for SUI. The FDA also recognizes that many patients are satisfied even if they only experience a reduction in urine leakage. Accordingly, the FDA recommends defining the clinically meaningful level as improvement in pad weight or improvement in the number of incontinence episodes defined as a reduction from baseline greater than 50%. Based on interim data of improvement in pad weight in the exploratory study, we selected the rate of patients with improvement in urine leakage volume (defined as reduction from baseline greater than 50% by 24-h pad test) at 52 weeks (last observation carried forward) as the primary endpoint of the ADRESU study.

The secondary endpoints are as follows:

1. Rate of patients at each evaluation time point with reduction in urine leakage volume greater than 50% from baseline by 24-h pad test
2. Urine leakage volume at each evaluation time point by 24-h pad test
3. Rate of patients at each evaluation time point with a reduction greater than 50% from baseline in the number of incontinence episodes per day
4. Number of incontinence episodes per day at each evaluation time point
5. Number of pads used per day at each evaluation time point
6. QOL score (International Consultation on Incontinence Questionnaire-Short Form [ICIQ-SF] and King’s Health Questionnaire [KHQ]) at each evaluation time point
7. Overall patient satisfaction at each evaluation time point
8. Urodynamic parameters (maximum urethral closing pressure, functional profile length, and abdominal leak point pressure) at each evaluation time point
9. Blood flow at the injection site measured by transrectal enhanced ultrasonography at each evaluation time point
10. Injection site evaluated by pelvic magnetic resonance imaging (MRI) scan at each evaluation time point

We also plan confirm the improvement tendency on QOL scores in SUI patients who satisfy the primary endpoint (defined as ‘Responders’), that is, that a point estimation of reduction rate on ICIQ-SF score is greater than 0%.

### Number of subjects

Our specific hypothesis is that the rate of ‘Responders’ as a primary endpoint, is higher than a pre-specified threshold of 10% given the poor likelihood of natural recovery of incontinence in these subjects [1–5]. Conservatively, the expected effectiveness rate is 30% because the rate of ‘Responders’ on interim analysis is 33.3% at 6 months and 66.7% at 12 months. Using two-side testing at a 5% significant level, we estimated that a sample of 41 patients was needed to achieve a 90% power of detection based on statistical calculations. Accordingly, a sample size of 45 patients was set in the ADRESU study anticipating a 10% loss to follow-up.

### Statistical analysis

Analysis of this trial is based on an intention to treat principle. The primary analysis is performed in the Full Analysis Set that consists of all the enrolled subjects who injected ADRCs. The rate of ‘Responders’ for the primary endpoint and the secondary endpoints of 1) and 3) were estimated and their 95% confidence intervals are calculated using the Clopper-Pearson method. The descriptive statistics (e.g., mean, standard deviation, minimum, median, and maximum) of the secondary endpoints of 2) and 4)-10) are provided at each evaluation time point.

## Discussion

Radical prostatectomy for male prostate cancer or prostatic hyperplasia causes a urethral sphincter dysfunction that can lead to persistent urinary incontinence. The underlying causes are thought to be the reduction of skeletal and smooth muscle, decreased blood flow, and denervation at the sphincter. We focused on adipose-derived stem cells for the functional recovery of the urethral sphincter because adipose tissue has more abundant multipotent stem cells than the bone marrow. Our basic research revealed that cultured adipose-derived stem cells improved leak point pressure and the amount of smooth muscle cells in an SUI rat model [17]. These promising basic research results drove us to implement a clinical exploratory study. However, when using cultured adipose stem cells, quality management of cell products is the hurdle in cell therapy for clinicians. The Celution System has a CE Mark approval in Europe, and it is a useful research tool for physicians. The Celution system is of particular interest to us for the application of this regenerative therapy. To the best of our knowledge, no previous studies have focused on the treatment of SUI with selected ADRCs prepared using the Celution systems. The interim result of this proof of concept clinical study for SUI patients showed the potential efficacy of ADRCs isolated by the Celution® system. As a new therapy for male SUI has not been developed in Japan as far as 2015, we aim to make our novel therapy widely available in Japan. As the following step, we discussed a development strategy of this novel therapy and the key design of the next pivotal study with the MHLW and PMDA. The MHLW suggested that we implement a clinical trial of the injection of ADRCs separated by the Celution® system as a new medical device based on the Pharmaceutical Affaires Act in Japan. Thus, we discussed the design of the investigator-initiated clinical trial (the ADRESU study) in a consultation meeting with PMDA. We constructed the rationale of the main design as a single-arm clinical trial for SUI in men based on several guidelines and previous research. As a result of a series of discussions in the consultation meeting, PMDA accepted our proposal.

### Ethics approval and current status on this trial

This study protocol is in accordance with the Declaration of Helsinki [26] and the Pharmaceutical Affaires Act in Japan. The protocol of this trial was approved by the following Institutional Review Boards: Nagoya University Hospital (No. 272001), Kanazawa University Hospital (No. 9009), Shinshu University Hospital (No. 1483), and Dokkyo Medical University Hospital (No. S-288). In June 2015, a clinical trial notification as a new medical device, much like a U.S. Investigational Device Exemption application, was accepted by the MHLW. The first patient completed the provisional registration in August 20, 2015 and received transplantation of ADRCs in September 1, 2015. The ADRESU study has currently enrolled 33 patients and is still recruiting patients from the four institutes (Nagoya University Hospital, Kanazawa University Hospital, Dokkyo Medical University Hospital, and Shinshu University Hospital). We are planning to enroll 45 patients as the full analysis set. The limit of enrollment will be December 2017, and the planned study end is March 2019.

## Conclusion

Herein, we present the overall design of this open-label, multi-center, single-arm study to evaluate the efficacy and safety of the periurethral injection of autologous ADRCs separated by the Celution® system in male SUI patients. In this article, we provide the key considerations regarding the overall study design, selection of subjects, selection of a control group, registration, response variables (endpoints), number of subjects and statistical analysis.

The ADRESU study is the first investigator-initiated clinical trial of a regenerative treatment for SUI patients using ADRCs separated by the Celution® system based on the Japanese Pharmaceutical Affaires Act. We will evaluate efficacy and safety in this trial to provide an adequate basis for marketing approval with the objective of disseminating this novel therapy to Japanese SUI patients. We believe that this translational medicine approach, often referred to as ‘from benchside to bedside to community’, has given rise to a novel treatment alternative for SUI in Japan.

## Abbreviations

ADRCs: Adipose-derived regenerative cells
FDA: Food and Drug Administration
ICIQ-SF: International Consultation on Incontinence Questionnaire-Short Form
KHQ: King’s Health Questionnaire
MHLW: Ministry of Health, Labour and Welfare
MRI: Magnetic resonance imaging
PMDA: Pharmaceuticals and Medical Devices Agency
PSA: Prostate specific antigen
QOL: Quality of life
SUI: Stress urinary incontinence
